# CASE REPORT Pan-Suture Synostosis After Posterior Vault Distraction

**Published:** 2013-10-03

**Authors:** Katrina F. Chu, Stephen R. Sullivan, Helena O. Taylor

**Affiliations:** ^a^Warren Alpert Medical School of Brown University; ^b^Department of Plastic Surgery, Rhode Island Hospital and Hasbro Children's Hospital, Providence, RI

**Keywords:** delayed craniosynostosis, distraction osteogenesis, secondary craniosynostosis, posterior vault distraction, Saethre-Chotzen syndrome

## Abstract

**Objective:** Posterior vault remodeling by distraction osteogenesis is a relatively new technique used for initial correction of turribrachycephaly in children with bicoronal craniosynostosis. We present a new potential complication from this procedure; a case of pan-suture synostosis subsequent to posterior vault distraction. **Methods:** We report an infant girl who presented with bicoronal synostosis in the setting of Saethre-Chotzen syndrome. She underwent posterior vault distraction and was distracted a total of 34 millimeters, with successful osteogenesis at the site. **Results:** One year postoperatively, the patient was found to have incidental, asymptomatic pan-suture synostosis on computed tomography. **Conclusions:** To our knowledge, this is the first reported case of delayed craniosynostosis after posterior vault distraction in the literature. The possible pathogenesis and significance of this case are discussed with a review of the current literature.

Surgical correction is advocated for both idiopathic and syndromic craniosynostosis to expand the cranial vault, relieve elevated intracranial pressure, prevent constriction of the synostotic skull on the developing brain, and correct cranial morphology. Posterior vault remodeling via distraction osteogenesis is increasingly used for the initial correction of turribrachycephaly^1^^-^4 in children with bicoronal synostosis, in which calvarial growth is limited in the anterior-posterior plane, and compensation occurs in the cranial-caudal direction. The relative benefit of posterior vault distraction over conventional cranial remodeling is controversial.^1^^,^^4^

Described complications of vault distraction include infection, hematoma, dural or cerebral spinal fluid leaks, and hardware complications.^5^ We present a new potential complication from this procedure; a case of pan-suture synostosis subsequent to posterior vault distraction. The possible pathogenesis and significance of this case are discussed with a review of the current literature.

## CASE REPORT

**Figure 1:**
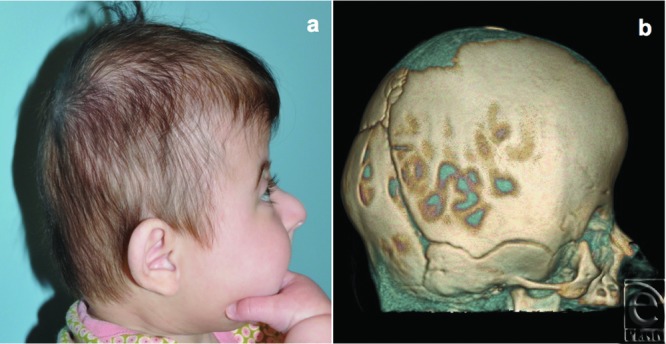
Three-month-old female patient with Saethre-Chotzen syndrome and bicoronal synostosis resulting in significant turribrachycephaly (*a*). The sagittal, lambdoid, and squamosal sutures were patent with a large fontanelle, characteristic of Saethre-Chotzen syndrome. Initial computed tomographic volume rendering shows bicoronal craniosynostosis (*b*).

**Figure 2:**
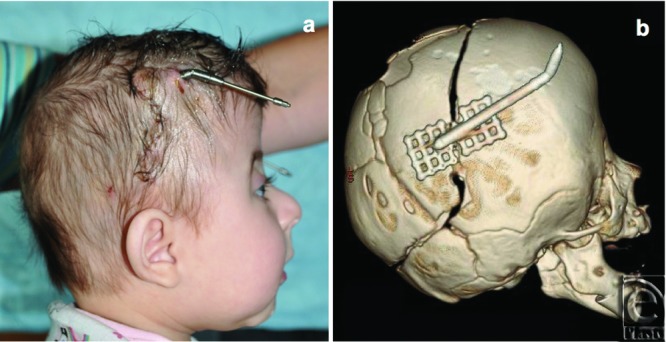
Bilateral craniectomy was performed and distraction plates connected to external arms were implanted along the osteotomy. At home, the family turned the arms 1 mm per day totaling 34 mm over 1 month.

**Figure 3:**
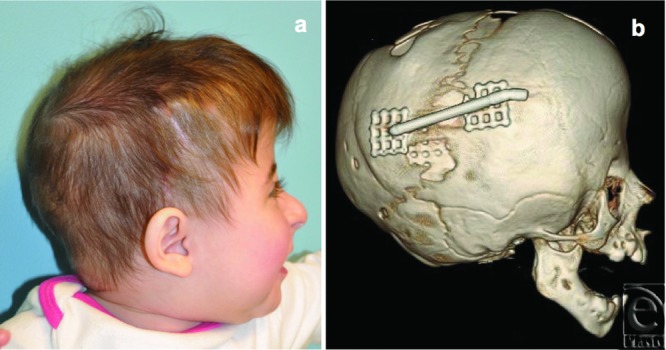
Clinical (*a*) and radiographic appearance (*b*) after 3 months of bony consolidation. Distraction hardware was subsequently removed.

Our patient presented at 3 months of age with bilateral coronal synostosis and profound turribrachycephaly (Fig 1). Gene testing was positive for *TWIST1* mutation, consistent with Saethre-Chotzen syndrome. She underwent bilateral posterior occipital craniotomy and posterior vault distraction at 6 months of age to lower the cranial high point and expand the anterior-posterior cranial length. Internal distractors with 4-cm distraction arms were placed along the osteotomy sites of the parietal bones; the intended vector was posterior-inferior from the skull apex (Fig 2). She tolerated the procedure well with a benign postoperative course. The patient's family was instructed to turn the distraction arms 1 mm per day at home. She was distracted 34 mm over 4 weeks, with correction of her occipital head shape and successful osteogenesis at the distraction site (Fig 3).

**Figure 4:**
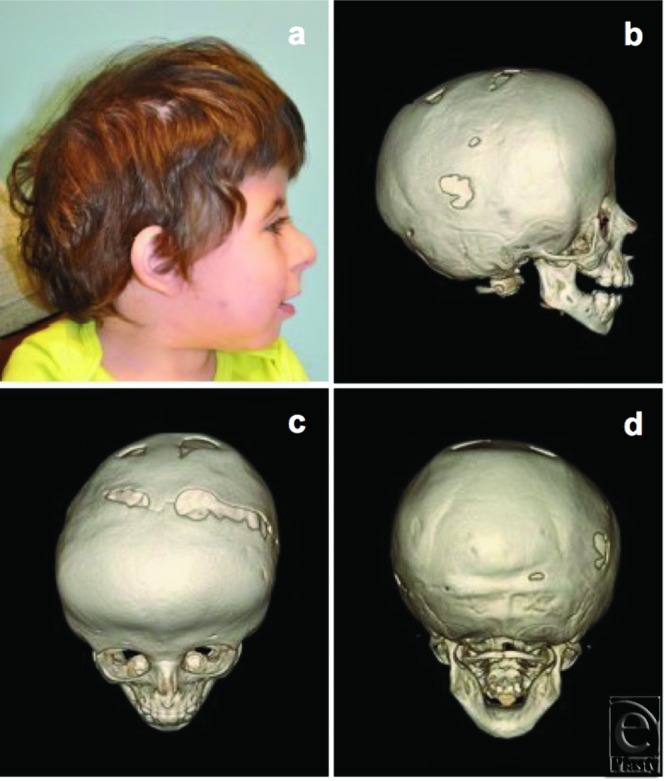
Clinical (*a*) and radiographic appearance (*b-d*) 1 year after posterior vault distraction reveals pan-suture synostosis. Routine computed tomography demonstrated interval closure of the sagittal and lambdoid sutures.

One year postoperatively, a computed tomographic scan revealed fusion of all her cranial sutures—a delayed pan-suture synostosis (Fig 4). Clinically, she had no evidence of increased intracranial pressure. Subsequent fronto-orbital advancement at 22 months improved forehead contour and brow position, and provided further increase in intracranial volume. To date, her postoperative course has been uneventful, without papilledema, headaches, or developmental delays.

## DISCUSSION

To our knowledge, this is the first reported case of delayed pan-suture craniosynostosis after posterior vault distraction. Whether the pan-suture synostosis was caused by the distraction procedure, or whether it was part of the underlying bony pathology of her syndromic craniosynostosis remains unclear. Secondary synostosis has been reported following strip craniectomies for synostosis^6^ as well as seemingly unrelated procedures such as hemispherectomy.^7^ Interactions between the dura mater and overlying sutures have been shown to affect cranial development,^8^^,^^9^ and one postulated mechanism in the literature is that interruption of these inhibitory signals on sutures may permit early fusion.^6^^,^^10^^,^^11^ This mechanism seems less likely in our patient's case, as the dura is not stripped from the native sutures in a distraction procedure.

Alternatively, mechanical forces may induce an epigenetic change that induces early suture fusion.^12^ Compressive forces in utero, secondary to twin gestation,^13^ or low pelvic station^14^^,^^15^ have been implicated in craniosynostosis. Animal models have capitulated the theory that compressive forces may induce premature fusion.^12^^,^^16^ In addition, it has recently been noted that helmet molding in infants after strip craniectomy for sagittal synostosis may induce secondary synostosis.^17^

It is also conceivable that our patient may have presented to us early in an eventual pan-suture process. This “progressive postnatal craniosynostosis”^18^^-^20 has been described in cases of Crouzon, Apert, and Saethre-Chotzen syndromes. However, given the timing of her synostosis shortly after her distraction procedure, we advocate careful monitoring and reporting of delayed synostosis after posterior vault distraction to better understand this phenomenon and prevent possible sequelae from undetected secondary synostosis.
